# Educating professionals to support self-management in people with asthma or diabetes: protocol for a systematic review and scoping exercise

**DOI:** 10.1136/bmjopen-2016-011937

**Published:** 2016-10-28

**Authors:** Nicola McCleary, Amanda Andrews, Susan Morrow, Sharon Wiener-Ogilvie, Monica Fletcher, Liz Steed, Stephanie J C Taylor, Hilary Pinnock

**Affiliations:** 1 Asthma UK Centre for Applied Research, Usher Institute of Population Health Sciences and Informatics, University of Edinburgh, Edinburgh, UK; 2 Education for Health, Warwick, UK; 3 NHS Education for Scotland, Edinburgh, UK; 4 Blizard Institute, Queen Mary University of London, London, UK

**Keywords:** Diabetes Mellitus (Types 1 and 2), Professional education, Self-management, Systematic review, Implementation

## Abstract

**Introduction:**

Supported self-management for asthma helps people adjust their treatment in response to symptom changes. This improves day-to-day control and reduces the risk of asthma attacks and the need for emergency healthcare. However, implementation remains poor in routine clinical practice. This systematic review is part of a programme of work developing an intervention to help primary care practice teams embed self-management support into routine asthma care. The aim of the review is to synthesise the evidence regarding the effectiveness of educational interventions for professionals supporting self-management in people with asthma or diabetes (type 1 and type 2). These two conditions have the most robust evidence base for the effectiveness of implementing supported self-management.

**Methods and analysis:**

Electronic searches will be conducted in CENTRAL, MEDLINE, EMBASE, ISI Web of Science, CINAHL, PsycINFO, AMED, Global Health, WHO Global Health Library, ERIC, BNI, RDRB/CME and Google Scholar. Eligible studies are randomised controlled trials or controlled clinical trials published between 1990 and 2016 which evaluated professional education interventions facilitating asthma or diabetes supported self-management. Further relevant work will be identified from trial registries, citation searching and through contact with authors of included studies. This will be supplemented by scoping potentially relevant educational packages described in English language policy literature or health service websites. Screening, data extraction and risk of bias assessment (using the Cochrane Risk of Bias Tool) will be completed by two independent reviewers, with a third reviewer arbitrating where necessary. We plan a theoretically informed narrative synthesis of the aggregated data as heterogeneity is likely to preclude meta-analysis.

**Ethics and dissemination:**

Ethical approval is not required for this systematic review. The results will be described in a paper submitted for peer-reviewed publication and will inform the development of an implementation intervention.

**Study registration number:**

PROSPERO CRD42016032922.

**Strengths and limitations of this study**

- Comprehensiveness of the synthesis will be ensured through searching a wide range of databases, performing prospective and retrospective citation tracking, checking trial registries and contacting authors of included studies.
- The systematic review will be supplemented with a scoping exercise of health service websites and related resources to ensure that initiatives introduced by healthcare services that are not in the published literature are incorporated into the synthesis.
- The review is limited by the high likelihood of heterogeneity precluding quantitative synthesis.
- Findings related to effective educational strategies will inform a whole systems intervention aiming to facilitate primary care practice teams to embed supported self-management into routine asthma care.

## Introduction

Asthma is common (affecting an estimated 5.4 million people in the UK) and responsible for unscheduled consultations, hospital admissions and ∼1000 deaths a year in the UK.^1^ Much of this morbidity is preventable with appropriate, timely self-management.^2^ ^3^ Regular structured review between the patient and a healthcare professional contributes to assisting the individual to effectively control their asthma,^4^ a concept described as ‘supported self-management’.^2^ ^3^ Though widely accepted definitions include supporting patients to ‘deal with the medical, role and emotional management of their conditions’,^5^ supported self-management in asthma as recommended by guidelines^2^ ^3^ focuses narrowly on adherence to medication/monitoring and the early recognition/remediation of exacerbations, summarised in (written) personal asthma action plans (PAAPs).^4^ ^6–9^

The Practical Systematic Review of Self-Management Support for long-term conditions (PRISMS) project provided a comprehensive overview of the evidence base for supported self-management in 14 long-term conditions (LTCs).^10^ In the context of asthma, the quantitative meta-review identified 23 systematic reviews synthesising data from 261 unique RCTs encompassing a broad range of demographic, clinical and healthcare contexts and concluded that supported self-management reduces exacerbations and improves control and quality of life.^10^ The qualitative meta-review identified one review which highlighted that patients want a medically focused PAAP set within the broader concept of ‘living with asthma’.^11^

Implementation of supported self-management, however, remains poor in routine clinical practice. An Asthma UK survey estimated that only 24% of people with asthma currently have a PAAP.^12^ The National Review of Asthma Deaths identified lack of PAAPs as a key preventable factor in the deaths that they investigated.^13^ Identified barriers to implementing asthma self-management support are practical (eg, time, no available paper-based PAAPs);^14^ conceptual (eg, mismatch between professionals' focus on clinical action plans and the advice patients want about ‘living with asthma’)^11^ and organisational (eg, professional communication between nurses who provide self-management education and general practitioners (GPs) who treat exacerbations).^15^ The systematic review of implementation studies conducted as part of PRISMS concluded that integration into routine practice required a whole systems approach in which motivated, skilled professionals support activated, informed patients within an organisation that values, promotes and monitors supported self-management.^16^

We are undertaking preliminary work to develop, refine and evaluate the clinical and cost-effectiveness of a practice-based intervention to implement self-management support for asthma in routine clinical practice in a UK-wide cluster RCT. Self-management support is defined as a service intervention that aims to empower patients to be active decision-makers who deal with the emotional, social and medical management of their illness and improve their independence and quality of life.^5^ An educational package for professionals who support people with asthma to self-manage will form a key component of the whole systems approach. Professional education is a prerequisite for effective implementation of supported self-management and will not only need to address the skills required by the professionals providing self-management education (typically asthma-trained general practice nurses in the UK) but also other members of the primary healthcare team providing services for people with asthma (including GPs, reception staff, prescribing clerks and community pharmacists).^10^

The aim of this systematic review is therefore to inform the development of the educational package by synthesising the evidence regarding the effectiveness of educational interventions for professionals involved in supporting self-management. The review will focus on diabetes as well as asthma in order to go beyond existing interventions in asthma and learn from professional education approaches in another condition where self-management support is well evidenced and often incentivised as fundamental to care. These two conditions have the most robust evidence base for the effectiveness of implementing supported self-management.^10^ A comparison of self-management interventions in asthma and type 2 diabetes found that while interventions in asthma focused on halting development of symptoms, studies in diabetes focused on integrating regimens into patients' lifestyles: self-management support interventions for type 2 diabetes therefore tended to be broader than those for asthma.^17^ Additionally, education for professionals on how to support self-management is key for the success of type 2 diabetes self-management support.^10^ Consequently, there may be valuable lessons to learn for professional education in asthma self-management support through comparing and contrasting the literature for the two conditions.

## Methods and analysis

We will follow systematic review procedures described in the Cochrane Handbook for Systematic Reviews of Interventions.^18^ The Preferred Reporting Items for Systematic review and Meta-Analysis Protocols (PRISMA-P) checklist has been used to guide the reporting of this protocol.^19^ If amendments to the protocol are made, the description of each amendment will be reported along with the amendment number and date. The review started on 2 November 2015 and will be completed by 29 November 2016.

### Eligibility criteria

#### Participants

The target population is professionals providing care to people with asthma or type 1 or type 2 diabetes mellitus. This includes doctors, nurses and health educators. Since the overarching purpose of the review is to inform the development of a primary care-based team intervention, primary care practice teams (including clinicians and administrative staff) are of particular interest. In this context, a primary care practice team is a team of professionals working within a community-based practice to provide patient care. Others who may support self-management in this context (such as pharmacists or lay/peer educators) will be included if their role is integrated within a primary care practice team, but excluded if the intervention did not involve the primary care team.

#### Interventions

Interventions of interest are educational packages designed to train professionals and/or practice teams to provide education to or support self-management in people with asthma or diabetes. These can comprise 14 components (information about condition/management; information about resources; plan/medication provision; regular review; monitoring and feedback; adherence support; equipment provision; access to advice/support; training/rehearsal for: communication with healthcare professionals, everyday activities, practical self-management activities, psychological strategies; social support and lifestyle advice and support).^20^

#### Comparators

In most trials of educational interventions, the comparator will be ‘no education’, though some may compare components of an educational package (eg, different modes of delivery, such as online vs face-to-face). The nature of the control service will be noted and accommodated within the analysis.

#### Outcomes

As this is a review of implementation-level interventions (ie, interventions aimed at changing health outcomes through changes in clinical practice), the primary outcomes of interest are categorised into two levels: process-level outcomes and health outcomes. Process-level outcomes reflect professional behaviour change. The primary process-level outcomes are the proportion of people with asthma receiving PAAPs and the proportion of people with diabetes receiving structured education.

The ATS/ERS Task Force report on asthma outcome assessment recommended that health outcomes in trials should reflect measures of current control and future risk.^21^ To maintain consistency, we have applied this recommendation to the selection of primary health outcomes for asthma and diabetes in this review. The primary outcomes representing current control are markers of asthma control (asthma control questionnaire or similar validated questionnaire) and HbA1c level for diabetes. The primary outcomes representing future risk are the proportion of people with an unscheduled consultation for acute asthma deterioration (eg, out-of-hours/GP consultation/A&E/admission), and acute events related to diabetic control and necessitating urgent action (eg, hypoglycaemia/hyperglycaemia/diabetic ketoacidosis).

To ensure that our outcomes reflect the broad view of self-management support as encompassing the emotional, social and medical management of illness, secondary outcomes comprise behavioural/cognitive measures related to professionals (eg, improvement in communication skills, confidence, competence) and patients (eg, self-efficacy, empowerment, and activation) and other measures of control (eg, symptom free days) or future risk (eg, exacerbations/steroid courses). When extracting secondary outcome data, outcomes assessed using validated tools will be prioritised.

#### Study design

Randomised controlled trials and controlled clinical trials will be included, since educational interventions may not always be evaluated in randomised controlled trials.

#### Setting

Any healthcare setting is of interest, though trials implemented within primary care teams will be of particular interest.

#### Years considered

Studies published from 1990 onwards will be included, as evolving professional educational approaches mean that earlier literature is unlikely to be relevant.

#### Language

There will be no language restrictions for included studies: the literature will be translated where possible, and any literature that we are unable to translate will be reported.

### Information sources

Electronic searches will be conducted in CENTRAL, MEDLINE, EMBASE, ISI Web of Science, CINAHL, PsycINFO, AMED, Global Health, WHO Global Health Library, ERIC, BNI, RDRB/CME and Google Scholar for studies published from 1990 until 2016. For all included studies, reference lists will be scrutinised and prospective citation tracking will be performed to identify additional relevant studies, including any qualitative work associated with included studies that may be helpful for providing further insights into our findings. We are not aware of any specific journals specialising in this literature which may require hand-searching: however, if such journals become apparent after gathering relevant studies, these will be hand-searched.

To identify relevant unpublished and in-progress studies, key internet-based relevant databases will be searched (UK Clinical Research Network Study Portfolio; the meta Register of Controlled Trials, http://www.clinicaltrials.gov and http://www.controlled-trials.com). Relevant qualitative studies which inform educational interventions (eg, published alongside trials)^22^ will be retrieved. Authors of included studies will be contacted to (1) source further published or unpublished results and/or training manuals related to their study if available and (2) source other relevant published, unpublished or ongoing studies including any related qualitative work.

We will supplement the published literature review by undertaking a scoping exercise of existing potentially relevant packages in asthma and diabetes through: (1) searching English language policy literature and health service websites for information about improvement initiatives involving up-skilling practices/clinical teams to improve self-management; and (2) contacting the initiative leads for information about the packages.

### Search strategy

A sensitive search strategy has been developed following advice from a senior librarian (Marshall Dozier, University of Edinburgh) using the Ovid interface for MEDLINE (see online supplementary file). This will be adapted for searches in other databases.

10.1136/bmjopen-2016-011937.supp1 supplementary file

### Data management

Literature search results will be exported to EndNote Library, which will be used for de-duplication, study screening and overall management of the retrieved records. Microsoft Word will be used to develop a data extraction form, which will be piloted and refined before use. Data will be extracted and stored electronically. Multiple reports from the same study will be treated as a single study, but we will draw on and make reference to all relevant publications.

### Selection process

One reviewer (NM) will undertake an initial filter of duplicates and clearly irrelevant titles. Before title and abstract screening begins, two reviewers (NM and AA) and the joint project leads (HP and SJCT) will independently screen a sample of 100 titles and abstracts from the searches for inclusion according to the review criteria in order to clarify interpretation of inclusion/exclusion criteria and as a quality control check. Any disagreements will be resolved by discussion and consultation with the project team, if required. This process will be repeated on further samples of 100 titles and abstracts until the level of agreement between all reviewers is deemed satisfactory (≥90%). The two reviewers will then independently screen all titles and abstracts, selecting potentially eligible papers for full text screening. The full texts of all potentially eligible studies will be retrieved and independently screened by the two reviewers. Disagreements at both stages will be resolved by discussion or arbitration by a third reviewer (HP or SJCT) if necessary. If after the full text assessment it is still unclear whether a study fulfils the inclusion criteria, the study authors will be contacted by email for clarification: if this fails, the respective study will be listed as a ‘potentially relevant study’. The searching and screening processes will be summarised using a PRISMA flow diagram.^23^

### Data collection process

The two reviewers (NM and AA) will extract the main findings from each study onto the data extraction form. The form will be piloted on a subsample of included studies to ensure it is easily and consistently interpreted and captures all relevant information. Data extraction disagreements will be resolved by discussion, or arbitration by a third reviewer (HP or SJCT) if necessary.

### Data items

Data will be extracted relating to general study characteristics, participant characteristics, details of the intervention and control conditions, the relevant outcomes assessed and corresponding results and information for assessment of the risk of bias.

### Risk of bias in individual studies

The two reviewers (NM and AA) will conduct independent assessments of methodological quality and risk of bias using the Cochrane Risk of Bias Tool.^24^ Disagreements will be resolved by discussion or, if necessary, arbitration by a third reviewer (HP or SJCT). The resulting risk of bias in included studies will be used to evaluate the robustness of the findings.

### Data synthesis

Descriptive tables will be used to summarise the characteristics of included studies. Frameworks such as TIDieR (a template for reporting interventions)^25^ and/or the Theoretical Domains Framework (a validated framework that identifies domains of theoretical approaches to behaviour change interventions which has been applied retrospectively to published interventions)^26–28^ will be used to describe the interventions. On a practical level, in order to inform the development of the educational component for our proposed implementation intervention, we will also take into account any frameworks used by Education for Health in the development of their courses.

A detailed descriptive summary of studies will be compiled, including data under the headings of: setting (primary/secondary care); at whom the intervention is directed (individual professional, groups, practice teams); mode of delivery (group, individual, face-to-face, online); components (lectures, workshops, assignments, practical skills, mentorship); duration and intensity of education/mentoring, generic/disease focused, outcomes assessed, information about uptake and any information about fidelity. We may undertake some short telephone interviews with authors in order to enhance our understanding of the interventions.

On the basis of preliminary scoping work, it is anticipated that there will be substantial heterogeneity so that meta-analysis will not be appropriate. A narrative synthesis of the aggregate data will therefore be undertaken. This will be achieved by developing a matrix of what has been shown to be effective/ineffective and the elements of the educational package (including content, mode of delivery, duration, intensity). Depending on the available data, graphical techniques (eg, Harvest plots)^29^ may be used to illustrate key outcomes and their relationship to these elements.

Although the overall pool of included studies are likely to be heterogeneous in nature, meta-analyses may be appropriate for subsets of studies with limited heterogeneity. For example, Cochrane reviews of professional education approaches have found that process-level outcomes are more often evaluated than patient health outcomes:^30^ ^31^ meta-analyses of some process-level outcomes may therefore be possible. Where appropriate, random-effects meta-analysis models for subsets of studies will be used, to take into account potential heterogeneity between studies.^32^ Heterogeneity will be quantified using the I^2^ statistic.

Qualitative data will be used to enhance our understanding of participants' perceptions of the impact of participating in the educational intervention on their professional practice. Data from qualitative studies will be synthesised thematically.^33^ An overarching narrative synthesis of quantitative and qualitative findings will be undertaken.^34^ Depending on the extent of the literature available in the different disease areas, subgroup analyses may be undertaken according to the targeted professionals (doctor, nurse, practice team) and/or setting (primary/secondary care). The findings of the scoping exercise of existing potentially relevant packages in asthma and diabetes will be used to supplement those of the systematic review.

The multidisciplinary research team, the wider project team and the steering group will meet regularly to discuss the emerging findings and aid interpretation. The PRISMA checklist will be used to guide reporting of the review.^23^

## Ethics and dissemination

Ethical approval is not required for this study, given that it is a systematic review using data already in the public domain. This review will inform the educational component of a whole systems intervention that will help primary care practice teams embed supported self-management into routine asthma care. A paper describing the review will be submitted for peer-reviewed publication. The infrastructure of the Asthma UK Centre for Applied Research (AUKCAR) will be used to support innovative approaches to dissemination (eg, via social media and Science Festivals).

## Conclusions

While patient education, professional training and organisational support are all essential components of successful self-management support, they are rarely effective in isolation.^10^ Effective implementation is multifaceted and multidisciplinary: it involves engaging patients and training and motivating professionals within the context of an organisation which actively supports self-management.^10^ ^16^ This review will achieve clarity on educational strategies likely to be effective in enabling professionals to implement supported self-management in their clinical practice and will inform the development of an educational package which will serve as one component of a whole systems intervention aiming to embed supported self-management into routine primary care asthma management.
