# Supplementary material for: Educating professionals to support self-management in people with asthma or diabetes: protocol for a systematic review and scoping exercise
Source: BMJ Open. 2016 Oct 28;6(10):e011937. doi: 10.1136/bmjopen-2016-011937 (PMC5093360; doi:10.1136/bmjopen-2016-011937)
Supplement: supplementary file [file bmjopen-2016-011937supp.pdf]

SUPPLEMENTARY FILE

MEDLINE Search Strategy

1. Primary Health Care/ or Family Practice/ or General Practice/
2. (primary care or primary medical care or primary health care or primary healthcare or general practice or family practice).mp.
3. Health Personnel/ or Medical Staff/
4. ((healthcare or health care) adj3 (provider? or practitioner? or professional?)).mp.
5. Physicians/ or Physicians, Primary Care/ or Physicians, Family/ or General Practitioners/
6. (general practitioner? or medical practitioner? or physician? or clinician? or doctor? or GP?).mp.
7. Nurses/ or Nursing Staff/ or Nurse Practitioners/ or Family Nurse Practitioners/
8. (nurse? or practice nurse? or community nurse? or nurse practitioner?).mp.
9. Medical Secretaries/ or Medical Receptionists/
10. (secretar$ or reception$ or administrat$).mp.
11. Pharmacists/
12. pharmacist?.mp.
13. Health Educators/
14. health educator?.mp.
15. Patient Care Team/
16. ((primary care or primary care practice or health care or healthcare or medical care or general practice or family practice) adj3 team?).mp.
17. or/1-16
18. Education/ or Health Education/
19. (educat$ or train$).mp.
20. (skill? adj3 develop$).mp.
21. Education, Professional/ or Education, Continuing/
22. (professional development or CPD).mp.
23. ((interprofessional or inter professional or inter-professional) adj3 (educat$ or train$ or develop$ or skill?)).mp.
24. ((team? or group?) adj3 (educat$ or train$ or develop$ or skill?)).mp.
25. Education, Medical/ or Education, Medical, Continuing/
26. (continuing medical education or CME).mp.
27. Education, Nursing/ or Education, Nursing, Continuing/ or Nursing Education Research/ or Nursing Evaluation Research/
28. Education, Pharmacy/ or Education, Pharmacy, Continuing/
29. Quality Improvement/
30. (quality adj3 improv$).mp.
31. or/18-30
32. Disease Management/
33. disease management.mp.
34. Self Care/ or Self Administration/ or Self Medication/
35. (self-manag$ or selfmanag$ or self-car$ or selfcar$ or self-help or selfhelp or self-administrat$ or selfadministrat$ or self-monitor$ or selfmonitor$ or self-medicat$ or selfmedicat$).mp.
36. (self adj3 (manag$ or car$ or help or administrat$ or monitor$ or medicat$)).mp.
37. Quality of Health Care/
38. (quality adj3 (care or healthcare or health care)).mp.
39. Professional-Patient Relations/ or Physician-Patient Relations/ or Nurse-Patient Relations/

40. (patient? adj3 (relation$ or communicat$)).mp.
41. ((action or treat$ or car$ or written or manag$ or medicat$) adj3 plan$).mp.
42. ((self-manag$ or self manag$ or selfmanag$ or self-car$ or self car$ or selfcar$ or self-help or self help or selfhelp or self-administrat$ or self administrat$ or selfadministrat$ or self-monitor$ or self monitor$ or selfmonitor$ or self-medicat$ or self medicat$ or selfmedicat$ or self-treat$ or self treat$ or selftreat$) adj3 plan$).mp.
43. (exacerbat$ or attack?).mp.
44. asthma control test.mp.
45. Hospitalization/
46. hospitali?ation?.mp.
47. After-Hours Care/
48. (out of hours or out-of-hours or OOH).mp.
49. Office Visits/
50. ((office or hospital or emergency department or ED or A&E or A & E or "accident and emergency") adj3 (visit$ or refer$ or admission$)).mp.
51. ((care or service?) adj3 (utili?ation or use?)).mp.
52. Patient Education/
53. Blood Glucose Self Monitoring/
54. Hemoglobin A, Glycosated/
55. HbA1c.mp.
56. Hypoglycemia/ or Hyperglycemia/
57. Diabetic Ketoacidosis/ or Hyperglycemic Hyperosmolar Nonketotic Coma/
58. (hyperosmolar hyperglyc?emic nonketotic syndrome or DKA or HNNS or HONK).mp.
59. glyc?emic control.mp.
60. or/32-59
61. Asthma/
62. (asthma or wheez$).mp.
63. (antiasthma$ or anti-asthma$).mp.
64. Respiratory Hypersensitivity/
65. ((bronchial$ or respiratory or airway$ or lung$) adj3 (hypersensitive$ or hyperreactiv$ or allerg$ or insufficiency)).mp.
66. Bronchial Spasm/
67. Bronchoconstriction/
68. (bronch$ adj3 (constrict$ or spas$)).mp.
69. (bronchoconstrict$ or bronchospas$).mp.
70. bronchial hyperreactivity.mp.
71. respiratory sounds.mp.
72. Diabetes Mellitus/
73. diabet$.mp.
74. Diabetes Mellitus, Type 1/
75. ((diabet$ or dm) adj5 (typ$ adj3 (one or "1" or I))).mp.
76. Diabetes Mellitus, Type 2/
77. ((diabet$ or dm) adj5 (typ$ adj3 (two or "2" or II))).mp.
78. Insulin Resistance/
79. ((insulin or noninsulin or non-insulin) adj3 (resistan$ or depend$)).mp.
80. (DM or DM1 or DM2 or T1D or T1DM or T2D or T2DM or NIDDM or IDDM or MODY).mp.
81. glucose $tolerance.mp.
82. or/61-81
83. Pragmatic Clinical Trial/ or Clinical Trial/ or Randomized Controlled Trial/ or Controlled Clinical Trial/

84. randomi?ed controlled trial.pt.
85. controlled clinical trial.pt.
86. (randomi?ed or randomly).ti,ab.
87. trial.ti,ab.
88. group?.ti,ab.
89. or/83-88
90. 17 and 31 and 60 and 82 and 89
91. (letter or review or comment or editorial).pt.
92. 90 not 91
93. (Animals/ or Nonhuman/) not Humans/
94. 92 not 93

Note: a free-text term related to professional behaviour (prof$ adj3 behav$).mp.) was considered for inclusion in section two of the above search, which is focussed on educational interventions. However, it was not included because when added, it did not retrieve any records additional to those already retrieved.
